# Analysis of copy number variation in dogs implicates genomic structural variation in the development of anterior cruciate ligament rupture

**DOI:** 10.1371/journal.pone.0244075

**Published:** 2020-12-31

**Authors:** Emily E. Binversie, Lauren A. Baker, Corinne D. Engelman, Zhengling Hao, John J. Moran, Alexander M. Piazza, Susannah J. Sample, Peter Muir

**Affiliations:** 1 Comparative Orthopaedic and Genetics Research Laboratory, School of Veterinary Medicine, University of Wisconsin-Madison, Madison, Wisconsin, United States of America; 2 Department of Population Health Sciences, School of Medicine and Public Health, University of Wisconsin-Madison, Madison, Wisconsin, United States of America; 3 Department of Comparative Biosciences, School of Veterinary Medicine, University of Wisconsin-Madison, Madison, Wisconsin, United States of America; University of Memphis, UNITED STATES

## Abstract

Anterior cruciate ligament (ACL) rupture is an important condition of the human knee. Second ruptures are common and societal costs are substantial. Canine cranial cruciate ligament (CCL) rupture closely models the human disease. CCL rupture is common in the Labrador Retriever (5.79% prevalence), ~100-fold more prevalent than in humans. Labrador Retriever CCL rupture is a polygenic complex disease, based on genome-wide association study (GWAS) of single nucleotide polymorphism (SNP) markers. Dissection of genetic variation in complex traits can be enhanced by studying structural variation, including copy number variants (CNVs). Dogs are an ideal model for CNV research because of reduced genetic variability within breeds and extensive phenotypic diversity across breeds. We studied the genetic etiology of CCL rupture by association analysis of CNV regions (CNVRs) using 110 case and 164 control Labrador Retrievers. CNVs were called from SNPs using three different programs (PennCNV, CNVPartition, and QuantiSNP). After quality control, CNV calls were combined to create CNVRs using ParseCNV and an association analysis was performed. We found no strong effect CNVRs but found 46 small effect (max(T) permutation *P*<0.05) CCL rupture associated CNVRs in 22 autosomes; 25 were deletions and 21 were duplications. Of the 46 CCL rupture associated CNVRs, we identified 39 unique regions. Thirty four were identified by a single calling algorithm, 3 were identified by two calling algorithms, and 2 were identified by all three algorithms. For 42 of the associated CNVRs, frequency in the population was <10% while 4 occurred at a frequency in the population ranging from 10–25%. Average CNVR length was 198,872bp and CNVRs covered 0.11 to 0.15% of the genome. All CNVRs were associated with case status. CNVRs did not overlap previous canine CCL rupture risk loci identified by GWAS. Associated CNVRs contained 152 annotated genes; 12 CNVRs did not have genes mapped to CanFam3.1. Using pathway analysis, a cluster of 19 homeobox domain transcript regulator genes was associated with CCL rupture (*P* = 6.6E-13). This gene cluster influences cranial-caudal body pattern formation during embryonic limb development. Clustered genes were found in 3 CNVRs on chromosome 14 (*HoxA*), 28 (*NKX6-2)*, and 36 (*HoxD*). When analysis was limited to deletion CNVRs, the association was strengthened (*P* = 8.7E-16). This study suggests a component of the polygenic risk of CCL rupture in Labrador Retrievers is associated with small effect CNVs and may include aspects of stifle morphology regulated by homeobox domain transcript regulator genes.

## Introduction

Anterior cruciate ligament (ACL) rupture is a common and severe condition of the human knee that is prevalent in individuals who participate in sports [1]. The incidence of ACL rupture per 100,000 person years is estimated at 77–130, with an ACL reconstruction incidence of 43.5 in the USA [2, 3]. Societal costs are substantial, even with the preferred cost-effective treatment of ACL reconstruction [1]. Knee osteoarthritis (OA) is prevalent after ACL rupture, and radiographic progression of OA occurs over time [4]. If ACL rupture is combined with other knee injuries, prevalence of OA increases, and symptoms are worse [4]. A non-contact valgus hyperextension mechanism explains most ACL ruptures [5, 6]. There is a high risk of second ACL rupture by rupture of the contralateral ACL or rupture of an intra-articular graft [7].

ACL rupture is a complex disease determined with genetic and environmental risk [8, 9]. Preemptive identification of at-risk individuals, particularly in athletes, would be advantageous because of the high morbidity and societal cost associated with the condition. Modification of environmental factors could help reduce ACL rupture prevalence [10]. Woman are predisposed to ACL rupture [11]. Family history influences risk of ACL rupture, as an individual with ACL rupture is twice as likely to have a relative with ACL rupture [12]. Candidate gene studies have shown that risk of ACL rupture is influenced by a number of genetic variants, principally genes that influence ligament matrix homeostasis [8, 9]. However, past research provides conflicting data with various limitations in study design leading to risk of bias [8]. A genome-wide association study (GWAS) has been performed in a human sample population of 102,979 with 598 ACL rupture individuals. No single nucleotide polymorphisms (SNPs) were found to meet genome-wide significance for association with affected individuals [13]. In the domestic dog (*Canis lupus familiaris*), cranial cruciate ligament (CCL) rupture in the stifle is a common condition which has been studied using genome-wide association [14]. Human anatomic terminology (i.e.: ACL, knee, anterior-posterior) will be used going forward for consistency with other canine model papers.

ACL rupture is a common and debilitating condition in many breeds of dog [15] and is typically a non-contact injury [16]. While no animal model can perfectly mirror the biomechanics of the human knee, the canine knee joint has been established as a model for human knee pathology for several decades [17]. Although dogs have a quadrupedal gait, ACL rupture in the two species has many shared clinical features, such as a non-contact mechanism [5, 16], high risk of contralateral rupture [18, 19], bilateral knee OA [18, 20], risk effects from sex steroids [11, 16], shared candidate genes [8, 9, 21], and a shared environment, suggesting that genomic discovery studies in the dog model are highly relevant to human ACL rupture. Similar to humans, progressive ACL fiber tearing develops in the presence of knee synovitis in dogs [20, 22]. Decreased knee laxity associated with low estrogen/progesterone in the follicular phase of the menstrual cycle in human females [11] and effects from ovariohysterectomy in female dogs [16] affect ACL rupture risk. Epidemiological risk factors for canine ACL rupture include breed, neutering of both male and female dogs, age, obesity, conformation, and knee synovitis [16, 23–25]. Dog breed is the most important risk factor for disease initiation, as ACL rupture prevalence varies considerably amongst different breeds [15]. Prevalence in the Labrador Retriever is high at 5.79%, while prevalence in a low-risk breed, such as the Greyhound, is low at 0.5% [15]. Pedigree studies have estimated narrow sense additive genetic heritability at 0.27 in the Newfoundland and 0.28 in the Boxer [26, 27]. GWAS in dogs confirmed ACL rupture as a complex polygenic disease with loci on chromosomes 1, 3, and 33 in the Newfoundland [28].

Discovery of complex disease-associated allelic variants is enabled by GWAS. However, disease-associated variants identified by GWAS typically account for small increases in risk when the disease is polygenic. Large scale structural and organizational changes in chromosomes have an important role in genetic susceptibility to common complex disease and often provide a substrate for evolutionary change with the creation of new genes [29, 30]. Copy number variants (CNVs) are large segments of DNA ranging from kilobases (kb) to several megabases (Mb) in length that vary in copy number, when compared to a reference genome [31]. Some disease-associated variants, including CNVs, may go undetected by SNP array GWAS due to small effects, gene-gene interaction, or low probe coverage [32]. Due to challenges detecting multibase variants with SNP markers, SNP array GWAS can be limited at detecting disease-associated structural variations [33]. Dissection of genetic variation in complex traits can be enhanced by studying CNVs [33, 34]. CNVs are extensive throughout the human and canine genomes. There are 100-fold more base pairs affected by CNVs than by SNPs [31]. CNVs can have important biological effects by directly altering gene expression or affecting gene regulation [30].

The dog is an ideal model species for CNV studies [31, 34]. The evolutionary history of domestication and breed development is a unique example of intense artificial selection. For hundreds to thousands of years, dogs have been non-randomly bred, creating bottlenecks and strong selection for specific behavioral and physical characteristics [35]. Dogs are one of the most phenotypically diverse mammals. Studies have scanned the whole genome of several dog breeds looking for breed-specific CNVs that contribute to within-breed patterns of phenotypic variation. For example, a high-density (probe spacing ~1kb) array was used to genotype 61 dogs, 6 of which were Labrador Retrievers, screening the whole canine genome for breed specific CNVs [36]. Labradors were reported to have an average of 179 CNVs per individual, with the mean length of 90.9kb [36]. Another study genotyped 351 dogs using the Illumina CanineHD BeadChip genotyping array (170K SNPs, probe spacing ~13kb) validating CNV detection from commercial SNP arrays [37]. Both studies concluded that breed specific CNVs exist, and many overlap possible disease susceptibility genes [36, 37].

Despite the species-wide diversity in dogs, there is reduced genetic heterogeneity within breeds. Extensive breed-specific genetic patterns explain why many breeds are predisposed to specific diseases. CNVs in the canine genome occupy 4.21% and span over 400 genes [38]. Breed-specific disease phenotypes are known to be influenced by CNVs. A study in Rhodesian and Thai Ridgeback dogs discovered a CNV duplication on chromosome 18 that associated with the dorsal hair ridge [39]. This duplication contains three fibroblast growth factor genes (*FGF3*, *FGF4*, and *FGF19*) that regulate embryonic hair and skin growth. The dorsal hair ridge in Rhodesian and Thai Ridgeback dogs is similar to dermal sinus, a neural-tube defect, in human beings. A genome-wide study of segmental duplications in dogs mapped a CNV affecting the glucokinase (hexokinase 4) regulator (*GCKR*) gene [38]. In humans, a GWAS identified a significant SNP in *GCKR* that associates with increased susceptibility to type 2 diabetes [40]. Dogs are an excellent model organism to study genetic diseases because they are affected by many of the same diseases as humans and reduced within-breed genetic variation enhances the ability to discover underlying genetic mechanisms [32].

To further explore the genetic contribution to ACL rupture we undertook genome-wide detection and analysis of CNVs that associate with ACL rupture in a population of pure-bred Labrador Retrievers. Our aim was to determine whether genomic CNVs form part of the genetic contribution to the polygenic complex trait of ACL rupture. We found 46 small effect CNV regions (CNVRs) associated with case status. Genes contained within these CNVRs were found to cluster to a biologic pathway for homeobox domain transcript regulators that influence anterior-posterior body pattern formation during embryonic limb development.

## Materials and methods

### Ethics statement

All procedures were performed in strict accordance with the recommendations in the Guide for the Care and Use of Laboratory Animals of the National Institutes of Health and the American Veterinary Medical Association and with approval from the Animal Care Committee of the University of Wisconsin-Madison (protocols V1070 and V005463). Informed consent from each owner was obtained before participation in the study. Animals were recruited from the University of Wisconsin School of Veterinary Medicine.

### Dogs

The Labrador Retriever breed was selected because of its high prevalence of ACL rupture [15] and because it is a common breed. All participating dogs were client-owned purebred Labrador Retrievers with four generation pedigree information. Full siblings were excluded from the analysis. A genomic relationship matrix (GRM) was created using SNP data and a singular value decomposition was performed to obtain eigen values and vectors to draw a PCA plot using *gaston* [41].

An orthopaedic examination to assess knee stability was performed on every dog. Dogs phenotyped as cases had anterior drawer on examination, with ACL rupture confirmed during surgical treatment. Preoperative knee radiographs were collected from affected dogs if available. Control dogs had bilateral lateral knee radiographs taken in a standing position. Lateral knee radiographs were evaluated for the presence of osteophytes and increased soft tissue density in the location of the infrapatellar fat pad due to synovial effusion. These radiographic degenerative changes are characteristic of ACL rupture in dogs [42, 43].

Dogs were considered controls if they were ≥8 years of age, had a normal orthopaedic exam, and normal knee radiographs. Canine ACL rupture is an acquired disease with risk of rupture peaking between ages 2–8 years. Of the 5.79% of Labradors that are affected with cruciate rupture, less than 6% develop ACL rupture after 8 years of age [44].

### Radiographic measurement and analysis of proximal tibial slope and relative tibial tuberosity width

Proximal tibial morphology is known to influence risk of ACL rupture in dogs [45, 46]. Therefore, posterior tibial slope (PTS) and relative tibial tuberosity width (rTTW) were measured from lateral radiographs [45–47]. When the entire tibia was not present on the radiographic view, the PTS angle was estimated using an established regression method [47]. Groups were created to calculate median PTS and rTTW for dogs with just the *HoxA* CNVR deletion, the *HoxD* CNVR deletion, both *HoxA* and *HoxD* CNVR deletions, and all three *HoxA*, *HoxD*, and *NKC6-2* CNVR deletions. The case and control group median PTS and rTTW excluded any dog with a *HoxA*, *HoxD*, or *NKX6-2* CNVR deletion. A Mann-Whitney-Wilcoxon test was performed to detect significant differences between case and control group PTS and rTTW.

### Genotyping

DNA was isolated from blood (EDTA collection tube) or from a saliva swab (PG-100 oral swabs, DNA Genotek, Ottawa, ON, Canada). Dogs were genotyped using the Illumina CanineHD BeadChip, which contains 173,662 SNP markers with an average probe spacing of 13kb. CanineHD BeadChip SNP locations are annotated on the canine CanFam2.0 genome build.

### GenomeStudio

The cluster file encodes normalized expected intensity of green and red fluorescence for all 3 expected genotypes (AA, AB, and BB) for every SNP. The BeadChip manufacturer, Illumina, generates a standard cluster file by genotyping many samples that are representative of the genetic diversity of the species of interest. The standard canine cluster file provided by Illumina was created using a set of 352 dogs from 26 different breeds. It is recommended that a user-generated cluster file is created if the study population is unique and does not fit well to the standard cluster file positions [48]. Creating a user-generated cluster file improves the accuracy of CNV calling and decreases noise. Only 14 of the 352 dogs used to make the standard cluster file were Labrador Retrievers. Consequently, a user-generated cluster file was created using the Gentrain2 cluster generation algorithm. We used 164 phenotype-negative control samples from the purebred Labrador Retriever data set for this file. Newly re-clustered SNPs were zeroed when Call Freq <0.97, Rep Errors >2, P-P-C Errors >2, Cluster Sep ≤0.3, AA R Mean ≤0.2, AB R Mean ≤0.2, BB R Mean ≤0.2, 10% GC Score ≤0.3, Hex Excess >0.2, A/B Freq ≥0.4, AB T Mean <0.2 or >0.8, A/A Freq = 1 and AA T Mean >0.3, A/A Freq = 1 and AA T Dev >0.06, B/B Freq = 1 and BB T Mean <0.7, A/A or B/B Freq = 0 and MAF>0, MAF<0.05 and 0.998>Call Freq>0.99 [48]. A user-generated cluster file was saved and exported from GenomeStudio for all canine autosomes. Chromosomes X and Y were excluded from the analysis.

Data were loaded into GenomeStudio from the raw *.idat files, sample sheet, the Illumina provided manifest file for the Canine HD BeadChip, and the user-generated cluster file. Samples with a call rate of <98% were excluded. SNPs with a genotyping rate of <95% were excluded. Log R ratio (LRR) and B allele frequency (BAF) values for every autosomal SNP were calculated and exported for each sample. The LRR is a measure of normalized total signal intensity and BAF is a measure of allelic intensity ratios. LRR is expected to equal 0 for every SNP, while BAF is expected to equal 0, 0.5, and 1 [49]. When samples deviate from the expected LRR and BAF, CNVs can be detected.

### PennCNV

PennCNV is an algorithm that implements a hidden Markov model to assess the LRR, BAF, the distance between neighboring SNPs, and the population frequency of the B allele (PFB) to detect CNVs [50]. To improve CNV calling, a GC model file was created by calculating the GC content of the 1Mb sequence around each SNP (500kb each side). The PFB file was generated using each SNP marker’s BAF from the study population. A minimum of 3 consecutive SNPs had to be affected for a CNV to be called. Quality control was performed, and samples were excluded if standard deviation of LRR >0.3, B allele drift >0.01, <-0.05 waviness factor >0.05, and number of CNV calls >150. Adjacent CNVs were merged using the clean_cnv.pl with default settings.

### CNVPartition

CNVPartition v3.2.0 is a plug-in software that works within GenomeStudio. CNVPartition compares observed LRR and BAF to fourteen predicted different copy number scenarios created as a bivariate Gaussian distribution [51]. To increase CNV calling accuracy, GC content was corrected with linear regression. For CNV calling, a minimum of 3 consecutive SNPs was required. After CNV calling, quality control metrics were plotted and samples were excluded if confidence <35, the standard deviation of LRR >0.3, and B allele deviation >0.05.

### QuantiSNP

QuantiSNP 2.0 is an algorithm that incorporates the LRR and BAF in an Objective Bayes Hidden-Markov Model and uses a fixed rate of heterozygosity for every SNP [52]. GC files were made using the Data Integrator tool made available by University of California Santa Cruz (UCSC) Genome Bioinformatics. QuantiSNP outputs values of outlier rate, standard deviation of LRR, and standard deviation of BAF for each chromosome for every sample. Values were summed and totals were plotted to compare all individuals. Quality control cut-offs were determined to be the sum of the standard deviation of LRR >7.5, the sum of the standard deviation of BAF >3.7, and the sum of the outlier rate >0.09. All CNV calls with a Log Bayes Factor <10 were removed.

### ParseCNV

ParseCNV is a software package that creates CNVRs in a dynamic case-control study design by grouping CNVs that are close in proximity and have comparable significance [53]. When ParseCNV is run with permutations it performs a Fisher’s Yates Shuffle to permute the array of cases and controls in place for association analysis and reports max(T) permutation test *P*-values that are corrected for the number of tests based on the permutation distribution. The number of permutations specified are performed on each significant CNVR detected and each observed test statistic is compared against the maximum of all permuted statistics meaning the max(T) permutation *P*-value reflects the chance of seeing a test statistic that large, given the number of tests performed. The permutation scheme preserves the correlational structure between CNVRs and provides a less stringent correction for multiple testing where moderate values such as 0.05–0.01 can represent genome-wide significance. CanFam2.0 reference files were created to run the program with a canine data set. The quality-controlled output for each of the three calling algorithms was analyzed separately. ParseCNV was run with 10,000 permutations. A nominal significance threshold (max(T) permutation *P*<0.05) was used to select CNVRs for inclusion in a pathway analysis. For overlapping CNVRs detected by multiple algorithms, Benjamini-Hochberg false discovery rate correction [54] of the association *P*-value was performed.

### Pathway analysis and CNVR lift over

Of the CNVRs that met the nominal significance threshold (max(T) permutation *P*<0.05), the CNVR regions were converted from CanFam2.0 to CanFam3.1 using the UCSC lift-over tool because CanFam3.1 is the current annotated assembly of the canine genome. During the lift-over process, five CNVRs were unable to be transferred from CanFam2.0 to CanFam3.1 due to an error message regarding portions of the regions being partially deleted in the new build. One CNVR that generated an error message was able to be successfully lifted over when a new start location was assigned within 100bp of the ParseCNV generated location. The four other CNVRs start and end locations were able to be lifted over when just the start and end bp locations were converted as points in the genome rather than trying to convert the whole region. A gene list was constructed by identifying genes that fall within the significant CNVRs as listed in the CanFam3.1 build on the UCSC and Ensembl genome browsers. A functional annotation of the gene list was processed using Database for Annotation, Visualization and Integrated Discovery (DAVID, https://david.ncifcrf.gov/summary.jsp) [55]. To overcome limitations in the canine genome annotation and canine annotation enrichments, genes were converted to the human ortholog and were mapped with DAVID to *Homo sapiens*. DAVID is an analytic biologic database that looks for meaningful clusters in large gene lists. In addition, the gene list was divided into genes found on the duplication CNVRs and genes found on the deletion CNVRs. The subset of genes associated with duplication and deletion CNVs were also analyzed separately through DAVID. DAVID pathway *P*-values had Benjamini correction applied.

### qPCR

DNA that was available from dogs with detected CNV deletions in the homeobox pathway was analyzed compared to control samples by qPCR to assess relative copy number. For each CNVR, two control Labrador Retrievers without ACL rupture that did not have reported CNV calls overlapping the investigated CNVRs were used as control samples for qPCR. For each of the three CNVRs in the significant homeobox pathway, the individual CNV calls were mapped to detect CNV breakpoints. DNA locations used for primer design were selected to avoid repeat regions reported in CanFam3.1 and were centered in areas of maximal overlap, where all individuals with detected CNV deletions had overlapping deletions within the significant CNVR. Primers were designed using IDT primer design programs (https://www.idtdna.com/scitools/Applications/RealTimePCR/). Ethanol precipitation with sodium acetate was performed on all DNA samples previously stored in AE buffer. The qPCR was performed in triplicate in 15μl volume using SYBR Green PCR Master Mix (Applied Biosystems) in a ViiA 7 Real Time PCR system (Applied Biosystems) with 5ng of genomic DNA. Forward and reverse primer final concentrations were 300nM each. Serial dilutions were performed for each primer pair to evaluate PCR efficiency. Amplification was performed under the following conditions: one cycle at 58°C for 2 minutes, one cycle at 95°C for 10 minutes, 40 cycles at 95°C for 15 seconds, and 60°C for 1 minute. After each qPCR run, dissociation curve analysis was performed to assess qPCR specificity. Relative copy number was quantified using the 2^-ΔΔCt^ method [56]. A previously validated reference gene for canine studies, *C7orf28B*, was used to normalize against the averaged triplicate C_t_ values [57].

## Results

### Study population

**Fig 1:**
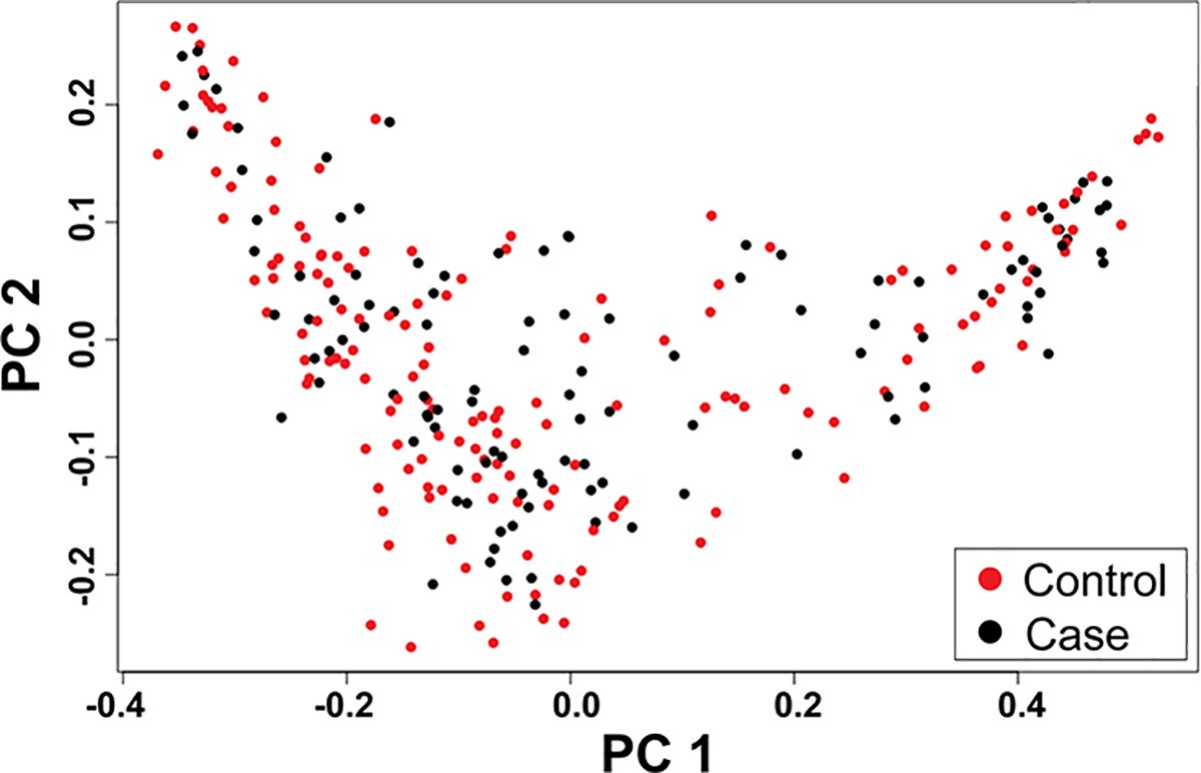
Principal Components Analysis (PCA) on 274 purebred Labrador Retrievers. Each dot represents an individual Labrador Retriever. PCA analysis was performed using *gaston* [41]. The 274 Labrador Retrievers included 164 phenotype-negative controls and 110 anterior cruciate ligament ruptured cases. The percent of total variance explained by principal component 1 was 33.76% and principal component 2 was 2.83%. The PCA plot reveals genetic homogeneity between the case and control groups.

Our purebred Labrador Retriever data set consisted of 164 phenotype-negative controls and 110 cases. PCA plot of 274 Labrador Retrievers showed genetic homogeneity between the case and control groups (Fig 1). Of the 274 dogs, 145 were male (90 controls, 55 cases) and 129 were female (74 controls 55 cases). The control group had a mean age of 10.5 years with a standard deviation of 1.75, while the affected group had a mean age of 6 and a standard deviation of 2.67. After genotyping, no individuals were removed due to low call rate. Quality control removed 30 dogs from the PennCNV dataset (148 controls, 96 cases), 17 from CNVPartition (154 controls, 103 cases), and 25 from QuantiSNP (144 controls, 105 cases). A total of 16 dogs were overlapping between the PennCNV and QuantiSNP excluded lists.

### CNVR association analysis

**Fig 2:**
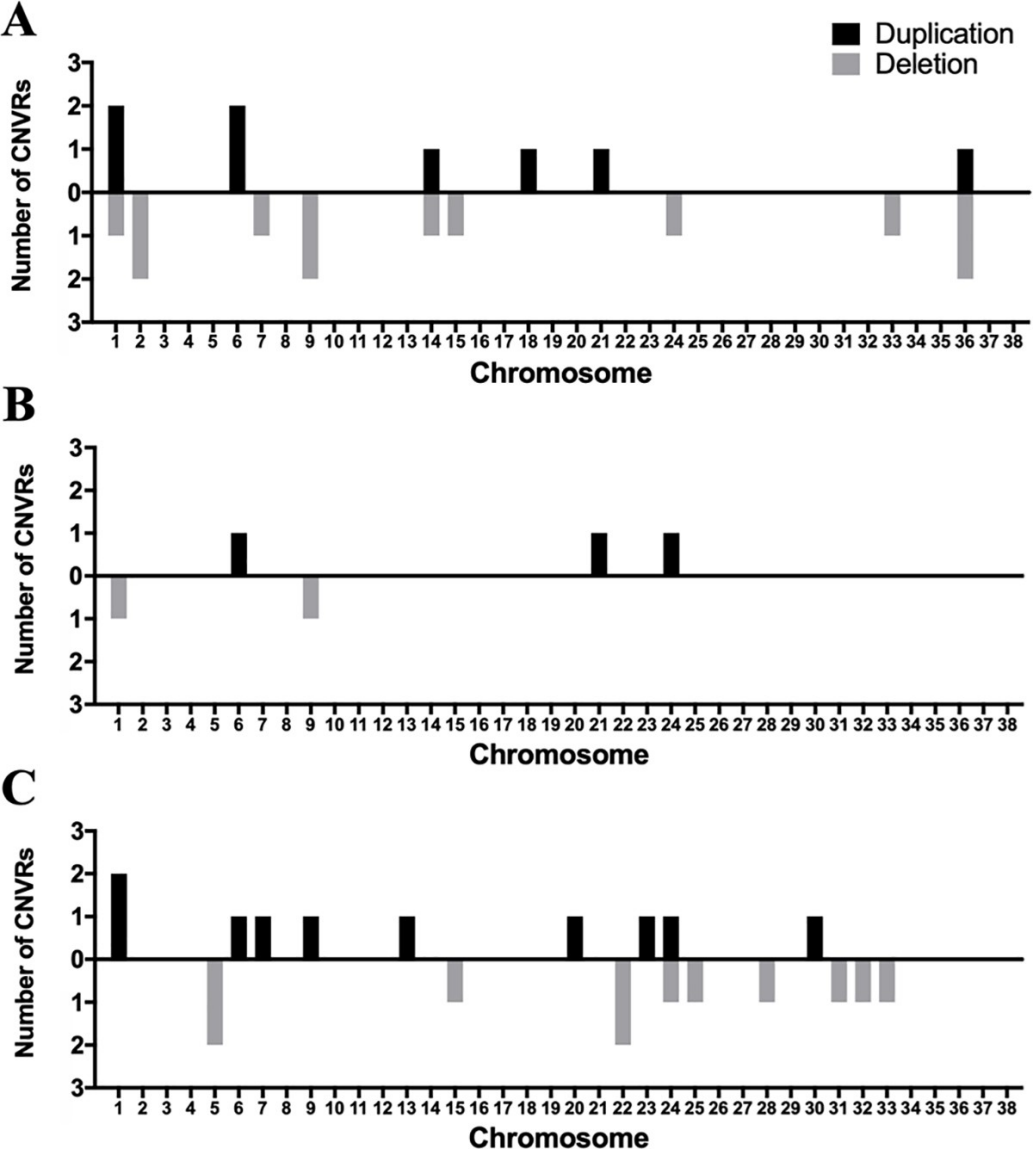
Association analysis of Copy Number Variant Regions (CNVRs) with canine Anterior Cruciate Ligament (ACL) rupture identified 46 associated CNVRs. CNVs were identified using three calling algorithms (PennCNV [50], CNVPartition [51], QuantiSNP [52]). Association analysis was performed using ParseCNV [53]. (A) Associated CNVRs detected by PennCNV. (B) Associated CNVRs detected by CNVPartition. (C) Associated CNVRs detected by QuantiSNP. Bars represent total deletion/duplication calls per chromosome. Associated CNVRs were found in 22 chromosomes.

Our analysis revealed no strong effect CNVRs and 46 small effect (max(T) permutation *P*<0.05) CNVRs that associated with ACL rupture. Frequencies in 42 of the associated CNVRs occurred in the population <10%, while 4 occurred at a frequency in the population 10–25%. CNVRs were identified on chromosomes 1, 2, 5, 6, 7, 9, 13, 14, 15, 18, 20, 21, 22, 23, 24, 25, 28, 30, 31, 32, 33, and 36 (Fig 2). Of these chromosomes, chromosomes 1, 6, 9, 21, and 24 had regions that were found by at least two of the calling algorithms. Regions on chromosome 6 and 24 were identified by all three algorithms. At the chromosome 6 location all three algorithms identified a duplication event that associated with cases. On chromosome 24, PennCNV and QuantiSNP called the region to be a deletion event that associated with cases, while CNVPartition identified a duplication event that associated with cases. Of the 5 CNVRs with multiple calls, all CNV associations remained significant after FDR correction.

**Fig 3:**
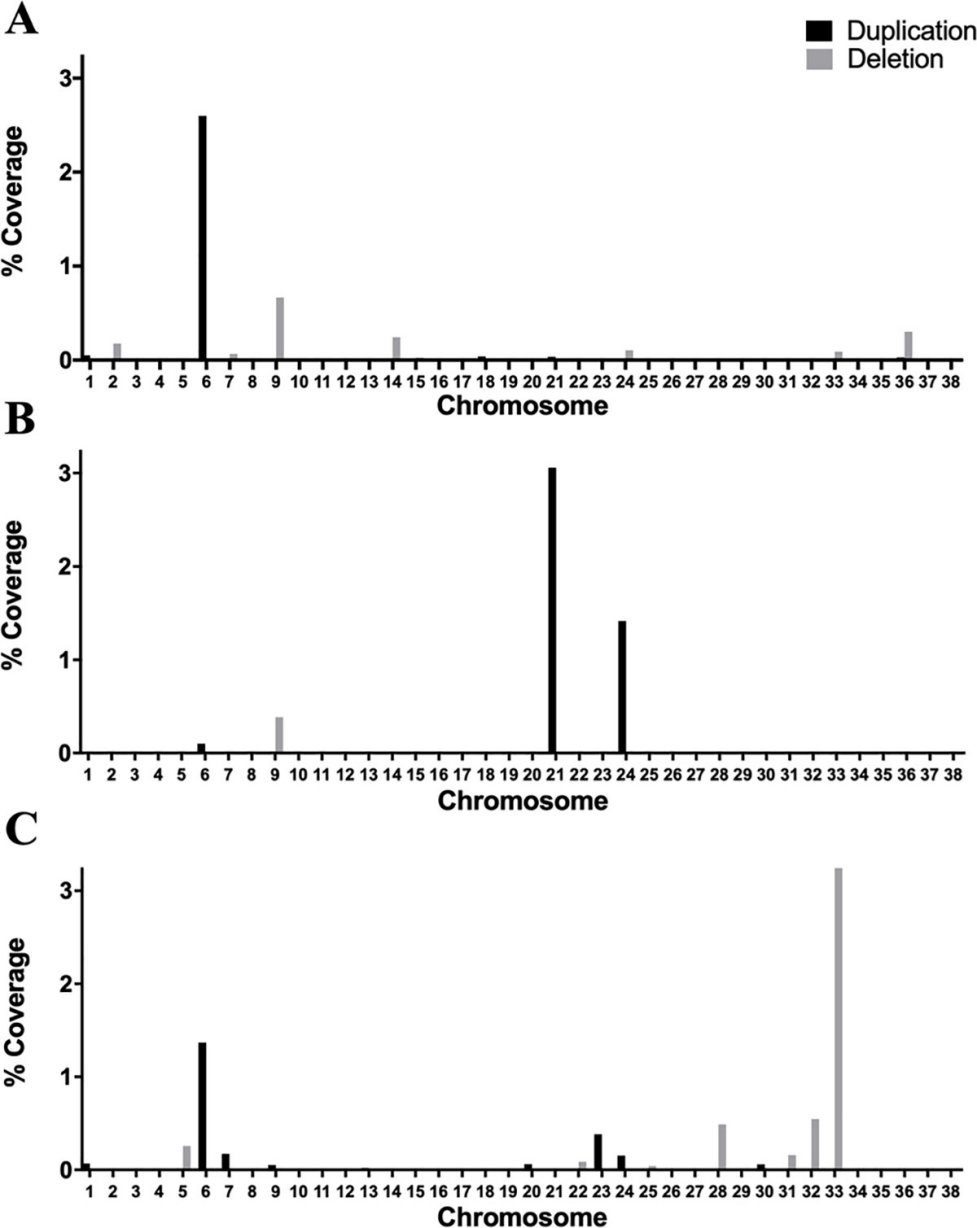
Percent coverage of the genome by Copy Number Variant Regions (CNVRs) that associate with canine Anterior Cruciate Ligament (ACL) rupture. CNVs were identified using three calling algorithms (PennCNV [50], CNVPartition [51], QuantiSNP [52]). Association analysis was performed using ParseCNV [53]. (A) Associated CNVRs detected by PennCNV. (B) Associated CNVRs detected by CNVPartition. (C) Associated CNVRs detected by QuantiSNP. Associated CNVRs were found in 22 chromosomes. Chromosome and genome percent coverage was calculated using the CNVR lengths generated from ParseCNV start and end bp output lifted over to CanFam3.1.

CNVRs were widely distributed across the genome. Of the 46 CNVRs identified, 25 were deletions and 21 were duplications. All of the 46 CNVRs associated with cases. The mean size for all CNVRs was 198872bp and the median 51348bp. CNVRs ranged in size from 7926 to 1557850bp. Chromosome 1 had the largest number of CNVRs of all the chromosomes. Chromosomes that had the highest percent base pair coverage by CNVs were 6, 21, and 33 (Fig 3).

**Fig 4:**
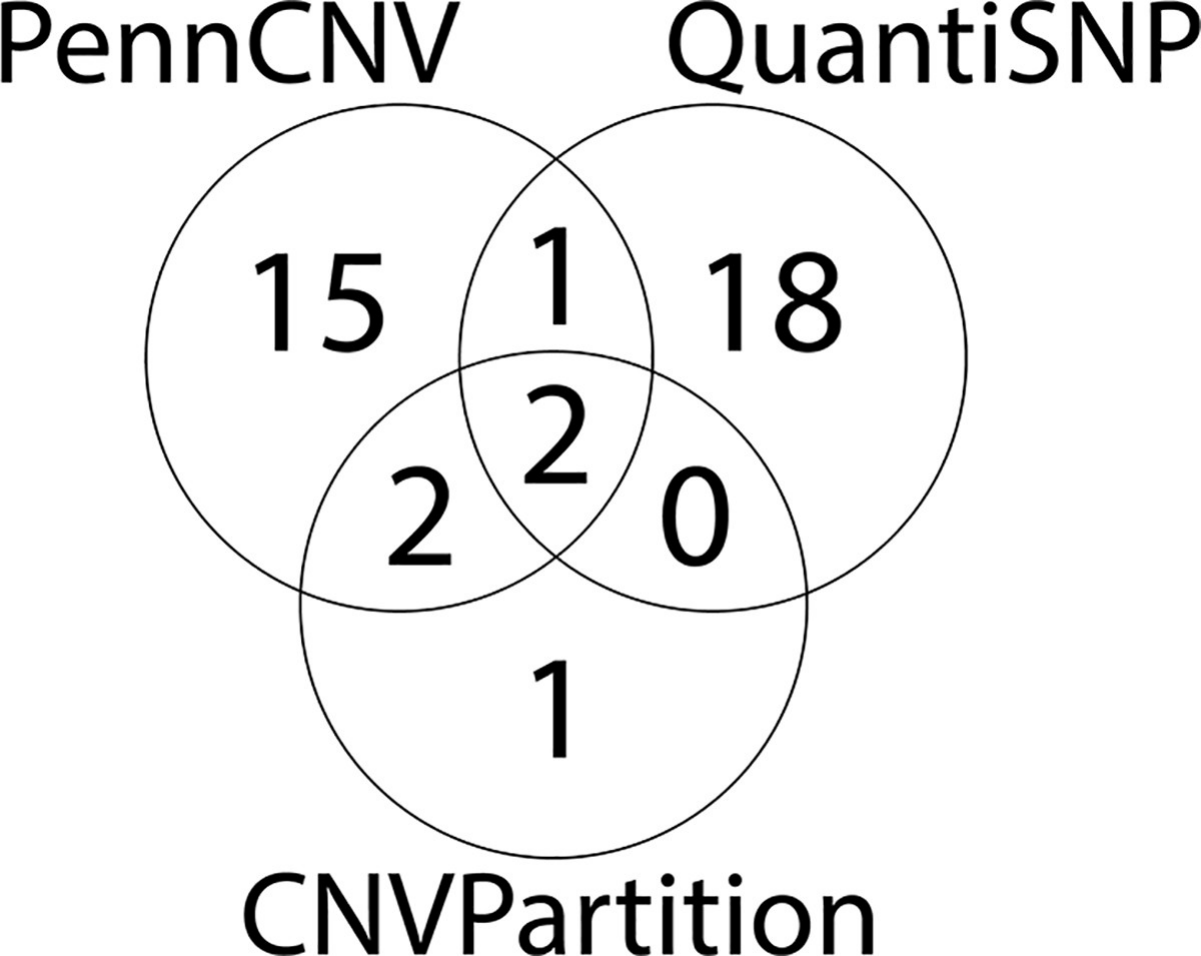
Overlapping Copy Number Variant Regions (CNVRs) from three CNV calling algorithms. CNVs were identified using three calling algorithms (PennCNV [50], CNVPartition [51], QuantiSNP [52]). Of the 46 significant CNVRs, 20 were called by PennCNV, 5 by CNVPartition, and 21 by QuantiSNP. There were CNVRs identified in 39 unique loci; 34 ACL rupture associated CNVRs were identified by a single calling algorithm, 3 were identified by two calling algorithms, and 2 were identified by all three algorithms.

The number of significant CNVRs called by PennCNV, CNVPartition, and QuantiSNP were 20, 5, and 21, respectively (Fig 4). CNVPartition detected the longest CNVRs with an average length of 511kb, while QuantiSNP had the shortest average length at 205kb. Conversely, QuantiSNP had the highest percentage coverage of the genome with significant CNVRs covering 0.15%, while CNVPartition had the lowest coverage at 0.11%. PennCNV performed in the middle for both length and percent base pair coverage with values of 259kb and 0.13%, respectively. From the 20 dogs that were randomly selected for concordance measurements, concordance between PennCNV and QuantiSNP had a mean of 53.4% with a standard deviation of 15.3, concordance between PennCNV and CNVPartition had a mean of 7.3% with a standard deviation of 9.4, and concordance between QuantiSNP and CNVPartition had a mean of 51.4% with a standard deviation of 15.

Many annotated genes were found within these CNVRs. None of the associated CNVRs overlapped with significant ACL rupture risk loci discovered previously in the same Labrador Retriever population GWAS [14]. In 46 CNVRs, a total of 152 genes were identified (Table 1). The chromosome 6 CNVR that was identified by all three algorithms contained the genes *RNPC3* and *AMY2B*. The chromosome 24 CNVR identified by all three algorithms contained the genes *OSBPL2*, *ADRM1*, and *RPS21*. Collagen genes *COL9A3* and *COL6A1* were within CNVRs on chromosomes 24 and 31, respectively. There were 12 CNVRs without any known genes in the CanFam3.1 assembly.

**Table 1:** Non-contact ACL rupture associated CNVRs identified in the Labrador Retriever using three CNV calling algorithms.

Chromosome	CNVR Start	CNVR End	Length (bp)	Max(T) Permutation *P*-value for Deletions that Associate with Cases	Max(T) Permutation *P*-value for Duplications that Associate with Cases	False Discovery Rate Corrected *P*-value for Overlapping CNVRs	Number of Cases with Deletion / Number of Diploid Cases	Number of Controls with Deletion / Number of Diploid Controls	Number of Cases with Duplication / Number of Diploid Cases	Number of Controls with Duplication / Number of Diploid Controls	Algorithm that Detected the CNVR	Genes	Papers Reporting Overlapping CNVRs
1	10,203,781	10,214,038	10,258	0.0254			4/78	0/123	0/78	0/123	CNVPartition		
1	11,323,575	11,368,842	45,268		0.0242		1/87	2/143	8/87	3/143	PennCNV		
1	14,563,023	14,575,286	12,264	0.0233			4/92	0/148	0/92	0/148	PennCNV	*KIAA1468*	
1*	16,281,556	16,337,721	56,166		0.0133	0.0133	0/100	0/144	5/100	0/144	QuantiSNP		Chen et al. 2009, Nicholas et al. 2011, Berglund et al. 2012
1*	16,320,005	16,337,721	17,717		0.0080	0.0133	0/91	0/148	5/91	0/148	PennCNV		Chen et al. 2009, Nicholas et al. 2011, Berglund et al. 2012
1	121,419,250	121,446,894	27,645		0.0221		1/98	2/141	6/98	1/141	QuantiSNP		
2	11,600,518	11,635,886	35,369	0.0270			10/84	5/137	2/84	6/137	PennCNV	*SKIDA1*	
2	85,122,962	85,238,711	115,750	0.0493			12/73	8/121	11/73	19/121	PennCNV	*CASZ1*, *APITD1*, *PEX14*	
5	32,207,352	32,374,761	167,410	0.0292			4/101	0/144	0/101	0/144	QuantiSNP	*CHRNB1*, *GABARAP*, *CTDNEP1*, *CLDN7*, *ELP5*, *YBX2*, *EIF5A*, *NEURL4*, *ACAP1*, *TNK1*, *PLSCR3*, *NLGN2*, *SLC2A4*, *ACADVL*, *GPS2*, *SPEM1*, *SPEM2*, *TMEM102*, *FGF11*	
5	60,282,278	60,342,624	60,347	0.0319			4/101	0/144	0/101	0/144	QuantiSNP	*ESPN*, *PLEKHG5*, *TNFRSF25*	
6	38,432,771	39,001,830	569,060		0.0013		1/88	1/147	7/88	0/147	PennCNV	*PDPK1*, *NTN3*, *ABCA3*, *E4F1*, *TRAF7*, *PKD1*, *ZNF598*, *RAB26*, *ECI1*, *GFER*, *PGP*, *NOXO1*, *CCNF*, *TBL3*, *RNF151*, *TSC2*, *CASKIN1*, *NDUFB10*, *DNASE1L2*, *MLST8*, *RPL3L*, *NTHL1*, *SEPX1*, *SLC9A3R2*, *ATP6V0C*, *TEDC2*, *RNPS1*, *BRICD5*, *MSRB1*	
6*	45,281,123	46,342,370	1,061,248		0.0043	0.0081	0/71	2/118	34/71	24/118	QuantiSNP		Chen et al. 2009, Nicholas et al. 2009, Nicholas et al. 2011, Berglund et al. 2012, Molin et al. 2014
6*	45,595,573	47,042,969	1,447,397		0.0054	0.0081	0/68	0/127	28/68	21/127	PennCNV	*AMY2B*, *RNPC3*	Chen et al. 2009, Nicholas et al. 2009, Nicholas et al. 2011, Berglund et al. 2012, Axelsson et al. 2013, Arendt et al. 2014, Molin et al. 2014, Ollivier et al. 2016
6*	46,949,172	47,027,777	78,606		0.0483	0.0483	0/59	0/104	36/59	37/104	CNVPartition	*AMY2B*, *RNPC3*	Chen et al. 2009, Nicholas et al. 2009, Nicholas et al. 2011, Berglund et al. 2012, Axelsson et al. 2013, Arendt et al. 2014, Molin et al. 2014, Ollivier et al. 2016
7	535,999	675,969	139,971		0.0322		0/101	1/143	4/101	0/143	QuantiSNP	*PPP1R12B*	
7	2,864,598	2,917,287	52,690	0.0023			9/87	1/147	0/87	0/147	PennCNV		
9*	732,580	997,748	265,168	0.0464		0.0464	6/89	2/144	1/89	2/144	PennCNV	*TMEM105*, *SLC38A10*, *ENTHD2*, *AZI1*, *AATK*, *BAIAP2*, *CEP131*, *NDUFAF8*, *TEPSIN*, *CHMP6*,	
9*	747,275	980,264	232,990	0.0401		0.0464	5/85	1/122	2/85	1/122	CNVPartition	*TMEM105*, *SLC38A10*, *ENTHD2*, *AZI1*, *AATK*, *BAIAP2*, *CEP131*, *NDUFAF8*, *TEPSIN*	
9	17,195,390	17,227,185	31,796		0.0173		1/90	0/137	14/90	7/137	QuantiSNP		Chen et al. 2009, Nicholas et al. 2009, Nicholas et al. 2011, Berglund et al. 2012, Molin et al. 2014
9	36,302,228	36,444,206	141,979	0.0161			11/84	5/143	1/84	0/143	PennCNV	*CA4*, *PSMG4*, *ZNHIT3*, *MYO19*, *PIGW*, *GGNBP2*, *USP32*	
13	35,849,962	35,862,869	12,908		0.0311		1/100	1/143	4/100	0/143	QuantiSNP	*PTP4A3*	
14	2,654,618	2,665,383	10,766		0.0257		3/79	7/132	14/79	9/132	PennCNV	*OR2T2*	Chen et al. 2009, Nicholas et al. 2011, Berglund et al. 2012, Molin et al. 2014
14	40,278,039	40,426,415	148,377	0.0158			11/85	5/141	0/85	2/141	PennCNV	*EVX1*, *HOXA3*, *HOXA4*, *HOXA5*, *HOXA6*, *HOXA7*, *HOXA9*, *HOXA10*, *HOXA11*, *HOXA13*, *MIR196B*	
15	8,078,846	8,088,532	9,687	0.0319			4/98	0/139	3/98	5/139	QuantiSNP	*CSMD2*	
15	9,007,530	9,024,348	16,819	0.0253			8/88	3/145	0/88	0/145	PennCNV	*CC2D1B*, *ZFYVE9*, *TUT4*	
18	42,199,134	42,221,726	22,593		0.0212		1/88	1/145	7/88	2/145	PennCNV	*RAPSN*, *PSMC3*, *SLC39A13*	
20	56,951,383	56,987,434	36,052		0.0222		3/92	6/134	10/92	4/134	QuantiSNP	*MOB3A*, *MKNK2*	
21*	45,012,402	46,568,089	1,555,688		0.0213	0.0426	0/65	0/104	21/65	16/104	CNVPartition	*LUZP2*	Nicholas et al. 2011, Molin et al. 2014
21*	45,012,402	45,030,442	18,040		0.0490	0.049	0/86	0/142	10/86	6/142	PennCNV		Nicholas et al. 2011, Molin et al. 2014
22	522,840	549,937	27,098	0.0026			7/97	0/141	1/97	3/141	QuantiSNP	*SERPINE3*, *INTS6*	
22	1,975,574	2,002,510	26,937	0.0050			6/99	0/144	0/99	0/144	QuantiSNP		
23	33,075,805	33,276,295	200,491		0.0306		0/101	1/143	4/101	0/143	QuantiSNP	*STAG1*, *SLC35G2*	
24*	46,256,338	46,306,925	50,588	0.0230		0.0345	4/92	0/148	0/92	0/148	PennCNV	*OSBPL2*, *ADRM1*, *RPS21*	Berglund et al. 2012
24*	46,306,925	46,982,353	675,429		0.0410	0.0410	1/88	3/134	5/88	1/134	CNVPartition	*OSBPL2*, *ADRM1*, *RPS21*, *COL9A3*, *CABLES2*, *GATA5*, *SLCO4A1*, *NTSR1*, *TCFL5*, *DIDO1*, *GID8*, *SLC17A9*, *YTHDF1*, *BIRC7*, *NKAIN4*, *RBBP8NL*, *MIR124-3*, *NTSR1*, *MRGBP*	Chen et al. 2009, Nicholas et al. 2011
24*	46,306,925	46,314,851	7,927	0.0126		0.0345	5/100	0/142	0/100	2/142	QuantiSNP	*OSBPL2*, *ADRM1*, *RPS21*	
24	47,484,099	47,557,195	73,097		0.0054		1/98	2/142	6/98	0/142	QuantiSNP	*PRPF6*, *SOX18*, *TCEA2*, *RGS19*, *OPRL1*	
25	17,983,587	18,005,251	21,665	0.0240			6/99	1/143	0/99	0/143	QuantiSNP	*GJA3*	
28	40,514,129	40,715,077	200,949	0.0493			5/99	1/139	1/99	4/139	QuantiSNP	*GPR123*, *ZNF511*, *NKX6-2*, *TTC40*, *INPP5A*, *CFAP46*	Nicholas et al. 2011
30	23,899,138	23,923,042	23,905		0.0139		0/100	0/144	5/100	0/144	QuantiSNP	*SLTM*	
31	39,364,930	39,428,602	63,673	0.0332			4/97	0/140	4/97	4/140	QuantiSNP	*PCBP3*, *FTCD*, *COL6A1*	
32	28,274,625	28,487,185	212,561	0.0291			4/101	0/144	0/101	0/144	QuantiSNP	*SGMS2*, *CYP2U1*, *HADH*	
33	551,701	580,231	28,531	0.0369			5/87	1/143	4/87	4/143	PennCNV		
33	607,740	1,625,465	1,017,726	0.0331			4/99	0/138	2/99	6/138	QuantiSNP	*EPHA3*, *PROS1*	
36	2,795,707	2,804,494	8,788	0.0353			5/91	1/147	0/91	0/147	PennCNV		
36	16,189,091	16,198,074	8,984		0.0213		6/86	7/141	4/86	0/141	PennCNV	*SLC25A12*	
36	19,876,345	19,978,344	102,000	0.0020			9/87	1/147	0/87	0/147	PennCNV	*EVX2*, *HOXD3*, *HOXD4*, *HOXD8*, *HOXD9*, *HOXD10*, *HOXD11*, *HOXD12*	

**Note:** Start and end CNVR positions and genes located within those regions are reported on the CanFam3.1 canine assembly. PennCNV [50], CNVPartition [51], and QuantiSNP [52], were used for CNV calling. Association analysis was performed using ParseCNV [53]. Max(T) permutation test *P*-values were calculated by ParseCNV using 10,000 permutations and were corrected for the number of tests based on the permutation distribution.

*Overlapping CNVRs confirmed by multiple algorithms. Benjamini-Hochberg false discovery rate correction [54] of *P*-values is also reported for overlapping CNVRs detected by multiple algorithms.

### Pathway analysis

DAVID grouped 19 genes into one homeobox domain significant biologic cluster. The Benjamini corrected *P*-value for the significant cluster was 6.6E-13. The 19 genes were *NKX6-2*, *EVX1*, *EVX2*, *HoxA3*, *HoxA4*, *HoxA5*, *HoxA6*, *HoxA7*, *HoxA9*, *HoxA10*, *HoxA11*, *HoxA12*, *HoxA13*, *HoxD3*, *HoxD4*, *HoxD8*, *HoxD9*, *HoxD11*, and *HoxD12*. These 19 genes were found in 3 CNVRs located on chromosomes 14, 28, and 36. *HoxA*, *HoxD*, and *NKX6-2* deletion CNVs were all identified by only one calling algorithm. PennCNV detected the *HoxA* and *HoxD* deletion CNVs while QuantiSNP detected the *NKX6-2* deletion CNV. The subset of genes found in duplication CNVRs grouped into a pathway of cell cycle, cell division, and mitosis regulators that was not significant. The subset of genes found in deletion CNVRs clustered the same 19 genes in a grouping with a lower Benjamini corrected *P*-value of 8.7E-16.

### Homeobox structural variation and knee morphology

PTS and rTTW values were measured from case and control radiographs. PTS and rTTW are quantitative parameters that describe morphology of the proximal tibia and have been shown to influence knee biomechanics by increasing load on the ACL, thus promoting ligament degeneration and fiber rupture [45, 58]. Radiographs were obtained for all dogs, but only a subpopulation of radiographs were determined to be of sufficient quality for measurement of PTS and rTTW, which included 125 controls (76%) and 57 cases (62%). Median PTS and rTTW were calculated for case and control groups and all dogs with any *HoxA*, *HoxD*, or *NKX6-2* CNVR deletion were excluded. The median PTS and range for the control and case groups was 28.0 (20.9–37.6) and 29.0 (20.9–35.0), respectively (Table 2). The median rTTW and range for the control and case groups was 0.70 (0.53–0.96) and 0.65 (0.45–1.00), respectively (Table 2). There was no significant difference between case and control group TPA. rTTW was significantly different between case and control groups (*P* = 3.0E-4).

**Table 2:** Homeobox CNVR deletion status and posterior tibial slope and relative tibial tuberosity width measured radiographically.

Dog Groups	PTS (degrees)	rTTW	Number of Dogs with Radiographic Measurements		Number of Dogs with Radiographic Measurements and Additional DNA for qPCR Validation	
			Controls	Cases	Controls	Cases
Controls^1^	28.0 (20.9–37.6)	0.70 (0.53–0.96)*	114			
Cases^1^	29.0 (20.9–35.0)	0.65 (0.45–1.00)*		56		
*HoxA* CNVR deletion	25.0 (22.0–28.1)	0.75 (0.56–1.00)	5	2		2
*HoxD* CNVR deletion	28.1 (28.0–28.1)	0.64 (0.63–0.83)	1	2		
*HoxA* and *HoxD* CNVR deletions	29.3 (25.0–31.7)	0.63 (0.62–0.71)		3		1
*HoxA* and *HoxD* and *NKX6-2* CNVR deletions	26.0	0.62		1		

**Note**: PTS and rTTW were measured from lateral knee radiographs [44–46]. Data represent median and (minimum-maximum).

^1^Control and case groups calculated PTS and rTTW excluded all dogs with any *HoxA*, *HoxD*, or *NKX6-2* CNVR deletion.

*rTTW was significantly different between cases and controls (*P =* 3.0E-4).

Of the dogs with radiographs that passed quality control, we found 7 (5 controls, 2 cases) dogs had *HoxA* CNVR deletions, 3 (1 control, 2 cases) dogs had *HoxD* CNVR deletions, 3 case dogs had both *HoxA* and *HoxD* CNVR deletions, and 1 case had CNVR deletions in *HoxA*, *HoxD*, and *NKX6-2*. PTS values in dogs with different types of homeobox CNVR deletions varied. PTS median and range was 25.0 (22.0–28.1) for dogs with *HoxA* deletions, 28.1 (28.0–28.1) for dogs with *HoxD* deletions, and 29.3 (25.0–31.7) for dogs with *HoxA* and *HoxD* deletions. Dogs with *HoxA* deletions had a median rTTW greater than the control group. Dogs with *HoxD* and *NKX6-2* CNVR deletions had lower rTTW values, similar to the case group.

### qPCR analysis of homeobox pathway CNVRs

**Fig 5:**
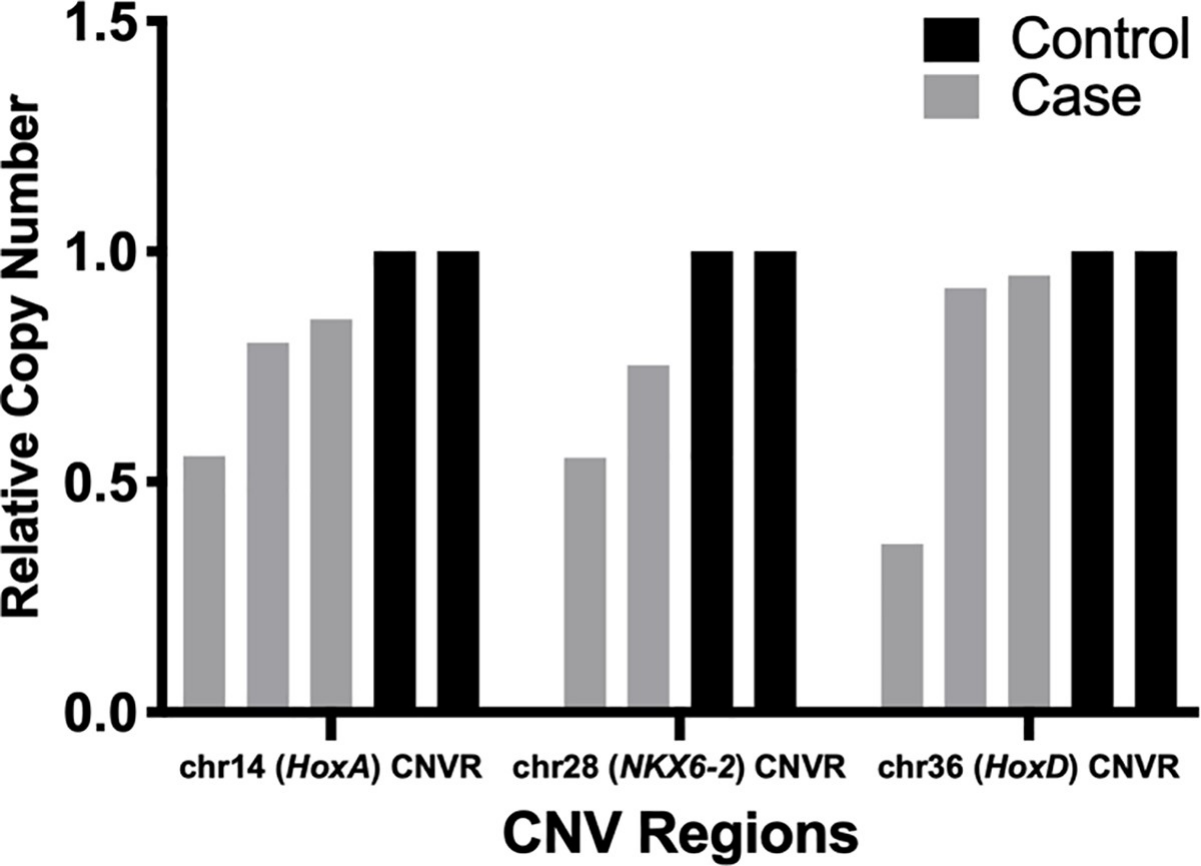
qPCR of homeobox pathway Copy Number Variant Regions (CNVRs). qPCR was performed to validate the CNVRs contributing to the biologic homeobox pathway. Primers were designed for each of the three CNVRs. Relative copy number was calculated by calibrating qPCR signal to an internal control gene (*C7orf28B* [57]) and then normalized to two Labrador Retrievers unaffected by ACL rupture with no CNV aberrations in the regions of investigation [56]. Reduction in relative copy number supports reduced copy number of genomic DNA in the CNVR of interest when compared to control individuals. CNV deletions were not validated with qPCR in 2 of the 3 dogs with *HoxD* CNVs called from SNP array data.

Of dogs with homeobox CNV deletions, additional DNA for qPCR analysis was available for 3 cases with *HoxA* (2 had radiographic measurements), 3 cases with *HoxD* (1 had radiographic measurements), and 2 cases with *NKX6-2* (none had radiographic measurements) CNVs. Quantitative PCR (qPCR) was performed to study the three CNVRs containing genes found in the significant homeobox pathway. For each of the three CNVRs, individual CNV calls were mapped to identify shared breakpoints. The chromosome 14 (*HoxA*) CNVR and chromosome 28 (*NKX6-2)* CNVR did not have distinct shared CNV breakpoints amongst individuals with detected CNV deletions. A method of relative quantification was used to estimate copy number at each location. Expected outcomes for a gene occurring in normal copy number of two would equal 1. A gene with a single copy loss would yield a relative copy change of 0.5. If a gene has two copies deleted, then the expected relative copy number would be 0. When compared to two control samples (2 Labrador Retrievers with no ACL ruptures and no reported CNV aberration in the region), the three dogs with the *HoxA* CNVR deletion had relative copy numbers of 0.55±0.009, 0.80±0.008, and 0.85±0.014 (Fig 5). The dog with a relative copy number of 0.55±0.009 for the *HoxA* CNVR deletion had a PTS of 25.0 and rTTW of 0.56 while the dog with a relative copy number of 0.80±0.008 in the *HoxA* CNVR deletion had a PTS of 22.0 and rTTW of 0.85. The dogs with *NKX6-2* CNVR deletions had qPCR relative copy numbers of 0.55±0.003 and 0.75±0.019. The third CNVR in the homeobox pathway containing the *HoxD* gene cluster had 3 affected dogs with reported deletions from the CNV calling that had qPCR relative copy numbers of 0.37±0.002, 0.92±0.012, and 0.95±0.013. Deletions were not validated with qPCR in 2 of the 3 dogs with *HoxD* CNVs called from SNP array data. No radiographic measurements were available on these 2 dogs. The affected dog with a validated *HoxD* decreased relative copy number (0.37±0.002) had a PTS of 29.3 and rTTW of 0.63.

## Discussion

The aim of this study was to identify candidate CNVs that contribute to the genetic risk of ACL rupture in dogs. By using one breed of dog, creation of a custom cluster file, and use of three different CNV calling algorithms, no strong effect CNVRs were identified. However, we found 46 small effect ACL rupture associated CNVRs, the majority occurring with <10% frequency in the population. Although the majority of CNVs have low frequency within this population of Labrador Retrievers they may represent independent *de novo* CNVs events. *De novo* CNVs are non-inherited sporadic structural mutations meaning CNVs that are present in offspring but are not present in their parents [33]. This form of non-inherited structural mutation occurring in a small number of affected individuals (<10) at the same genomic location or affecting similar biologic pathways could be very significant because the rate of sporadic structural mutation is much lower than the rate of CNV inheritance. Therefore, CNVs present in only a handful of cases can potentially imply subtle but strong disease association [33, 59]. In humans, there are several examples where low frequency *de novo* CNVs have been found to significantly contribute to complex polygenetic disease risk [60–63].

A list of 152 annotated genes from the associated CNVRs formed a significant cluster of 19 genes into a biologic pathway that regulates skeletal morphology. These 19 genes were located in 3 CNVRs identified by only one calling algorithm. PennCNV detected two of these CNVRs and QuantiSNP detected the other CNVR. This clustering was enhanced when genes associated with only CNV deletions were considered. This enriched pathway included homeobox genes of the *Hox* classes A and D. *Hox* genes encode transcription factors that are essential embryonic regulators of body patterning and limb formation. The vertebrate genome has 39 *Hox* genes that are separated into four distinct chromosomal clusters, *HoxA-HoxD* [64, 65]. *Hox* expression during embryogenesis is complex and precise timing, quantity, and spatial distribution is crucial for proper development [66]. Several human and mouse limb abnormalities have been linked to mutations and CNVs that alter *Hox* genes or their regulatory elements [67]. Specifically, *HoxA* and *HoxD* have been reported to be the most important clusters for proper limb development [68]. Conditions such as synpolydactyly and split hand/foot malformation have been shown to be a consequence of *HoxD* mutations [69].

Association of canine PTS and ACL rupture is controversial; some studies have linked a greater PTS with higher incidence of ACL rupture [45, 70]. In the dogs of the present study there was no significant difference between case (n = 56) and control (n = 114) group PTS. Smaller relative tibial tuberosity widths have been associated with ACL rupture in dogs [45], likely a consequence of increased anterior tibial thrust under load. In this study, affected dogs had significantly smaller rTTW than controls. The *HoxD* (n = 3), *HoxA* and *HoxD* (n = 3), and *HoxA*, *HoxD* and *NKX6-2* (n = 1) deletion groups had particularly low rTTW values. Our data suggest these CNVR deletions may play a role influencing tibial tuberosity width in a subset of ACL rupture dogs. However, these results should be interpreted with caution because of small sample size and wide range of measured values. Investigation of additional dogs to increase power and permit statistical analysis is needed to further understand these observations and confirm significance of these associations.

Tibial and femoral conformation is a risk factor for canine ACL rupture. Effects on knee joint incongruity, changes in articular contact areas, altered joint angles, or increased stress loading on the ACL may all be important [71, 72]. In human beings, lateral femoral condyle height, as well as anterior-posterior and medial-lateral tibial plateau distances, have been shown to significantly differ between people with and without ACL rupture [73]. Although *Hox* genes primarily control patterning and growth of skeletal elements, several studies have demonstrated *Hox* genes involvement in cartilage differentiation and synovial joint organization [74, 75]. *Hox* mutations can disrupt collagen fibril formation and extracellular matrix production that results in abnormal chondrogenesis by affecting downstream *Shh* and *Sox9* expression [75, 76]. Mechanisms that affect passive knee joint stabilizers (menisci, joint capsule, collateral ligaments), changes to ACL fibroblasts, collagen fibril abnormalities, or the synovial sheath have also been suggested to influence canine ACL rupture [71]. With *Hox* developmental pathways and their downstream effects being so widespread it is not possible to determine a specific mechanism associated with these CNV deletions that increase risk for ACL rupture without further research.

With only 18 affected dogs possessing homeobox CNVR deletions, reduced signaling of this pathway is not the only genetic mechanism influencing risk of canine ACL rupture. ACL rupture is a complex polygenic disease [14, 21, 28, 77]. Our results suggest alterations in *HoxA*, *HoxD* and *NKX6-2* expression may influence risk of ACL rupture in a proportion of dogs. Further investigation of bone and joint morphology is needed to robustly define and validate the relationship between *Hox* deletions and ACL rupture.

CNVs associated with ACL rupture likely influence multiple physiological pathways, as genes from the significant homeobox pathway represent only 3 of the 46 ACL rupture associated CNVRs. Areas of substantial interest for future investigation would include CNVRs with the largest base pair coverage per chromosome, CNVRs that are verified by multiple programs, and CNVRs containing genes associated with collagen matrix. The 2 CNVRs that had overlapping areas and confirmed by all 3 algorithms were located on chromosomes 6 and 24. Overlapping CNVRs have been reported in previous canine CNV discovery papers in these same regions supporting the observation that these genomic regions exhibit structural variation (Table 1). Of the overlapping regions on chromosome 6, 2 CNVRs contained the genes *AMY2B* and *RNPC3*. *AMY2B* encodes for pancreatic amylase. In the dog, *AMY2B* has historically been associated with increased copy number and domestication [78–80]. Domestic dogs have various increases in copy number of *AMY2B* when compared to wolves [79]. *AMY2B* duplications result in high amylase activity which increases starch digestion efficiency. More recent canine domestication research has identified a large duplication in the genomic region distal and separate from *AMY2B* that is centered on *RNPC3* [81]. *RNPC3* encodes for a small nuclear ribonucleoprotein (snRNP) that is a component of the minor U12-dependent spliceosome [82]. Spliceosomes regulate pre-mRNA processing and removal of non-coding sequences and introns. The U12-dependent spliceosome removes U12-type introns that represent less than 0.5% of all introns. Although U12-type introns are found at much lower frequencies, U12 type splicing is still essential for gene expression, but much of its importance is not yet characterized. Alterations to the U12-dependent spliceosome result in abnormal growth, developmental defects, and disrupted gene expression [83]. *RNPC3* has been linked to dwarfism in humans through defective pituitary somatotroph development and subsequent growth hormone deficiency [84]. It is unclear by what biologic mechanism a duplication CNV in *RNPC3* might influence risk for ACL rupture, although alterations to copy number of *RNPC3* in dogs potentially could result in global phenotypic effects on body size as suggested by its association to the canine domestication syndrome [81]. Two of the three overlapping regions were roughly 1Mb in length harboring only the *AMY2B* and *RNPC3* genes. It is also possible that this region on chromosome 6 is functioning as a regulatory region influencing expression of genes occurring at other chromosomal locations.

The CNVR on chromosome 24 was called by all three algorithms. *OSBPL2* appeared in all 3 regions and *COL9A3* was only found in one of those regions. *OSBPL2* is a lipid binding protein that is an important regulator of intracellular cholesterol homeostasis through lipid metabolism and transport. Knockout *OSBPL2* bama miniature pigs have hypercholesterolemia, increase in adipocytes, with obesity phenotypes [85]. The link between decreased *OSBPL2* expression and obesity in an animal model is interesting given obesity is a known epidemiological risk factor for canine ACL rupture and two of the chromosome 24 CNVRs were deletions. *COL9A3* encodes for the *α*3 chain of type IX collagen. In humans, mutations in *COL9A3* have been associated with two genetically and phenotypically heterogenous types of osteochondrodysplasia, multiple epiphyseal dysplasia (MED) and pseudoachondroplasia (PSACH) [86–88]. MED is clinically characterized by mild to moderate short stature, abnormally small and/or irregular epiphyses, and early-onset OA predominantly in the hip and knees. PSACH is characterized by disproportionate short-limbed short stature, joint laxity, long bone deformities and early-onset OA. Both MED and PSACH can have varying levels of phenotypic severity. Interestingly, *COL9A3* mutations have been linked to oculoskeletal dysplasia (OSD) in the Labrador Retriever [89]. OSD dogs can have a range of phenotypes which include short-limbed dwarfism, delayed epiphyses development, cataracts, retinal dysplasia, and retinal detachment [90]. One study performed candidate gene analysis for ACL rupture in the Newfoundland including *COL9A3* as a candidate gene because of its known associations with primary arthritis and osteochondrodysplasias in humans [91]. No significant association was found with *COL9A3* and ACL rupture in the population of 90 Newfoundland dogs. Another candidate gene study found no association with *COL9A3* and ACL rupture in 12 Boxers [92]. Despite the Newfoundland and Boxer representing high-risk ACL rupture breeds, the candidate gene approach is most successful at identifying large effect mutations associated with monogenic traits and is limited at complex disease small-effect variant discovery [93].

Although the CNVR on chromosome 31 containing *COL6A1* was only called by one program, *COL6A1* has interesting associations with tendon and synovial joint biomechanics and OA. The *COL6A1* gene encodes for the *α*1 chain of type VI collagen. Collagen VI is a non-fibrillar collagen found in extracellular matrix of muscle and most connective tissues. Mutations in *COL6A1* are most famously linked to a wide range of muscular dystrophies of varying severity in humans [94]. These collagen type VI disorders can have tendon involvement [94]. Normal tendon is typically composed of a few fibroblasts in a dense extracellular matrix of type I collagen fibrils with surrounding scattered collagen VI. In humans, severe myopathy caused by a collagen VI mutation is called Ullrich congenital muscular dystrophy (UCMD). Tendons of UCMD patients can have changes to pericellular matrix organization causing an abnormal accumulation of collagen type VI and altered distribution of collagen type I which results in dysfunctional fibrillogensis [95]. Subsequently, severely affected UCMD humans can have tendon fibrils with irregular profiles and decreased mean diameter. *COL6A1* knockout mice have similar tendon changes with disorganized collagen fibers with abnormal fibril structure and decreased fibril diameter [94, 96]. These mice have a ~2.5 increase in tendon fibril density which results in biomechanical changes with significant reduction in maximum load and stiffness [96]. *COL6A1* knockout mice also have significant changes to their trabecular bone structure with decreased bone volumes that have been noted in the proximal tibia [97]. These knockout mice also experience accelerated OA development due to loss of stiffness in the articular cartilage pericellular matrix revealing type VI collagen has a role in regulating the physiology of chondrocytes and physiology of the synovial joint [98]. A candidate gene study included *COL6A1* SNPs to investigate the genetics of ACL rupture in a population of 271 Newfoundlands, 289 Labrador Retrievers, 138 Rottweilers, and 51 Staffordshire Bull Terriers [21]. This candidate gene study found no significant associations between *COL6A1* and ACL rupture. However, advanced bioinformatic analyses in humans continues to find significant associations with OA and *COL6A1* in differentially expressed genes and pathways [99, 100].

This study found that CNVRs associate with cases, suggesting CNVs are not acting in a protective manner in control dogs, but rather are disrupting gene dosage or gene regulation to increase risk of ACL rupture. In theory, CNVs could similarly be found to associate with control status and offer adaptive advantages to decreased disease risk. However, the extensive amount of CNV disease association studies in humans and dogs supports the idea that non-adaptive or disadvantageous CNVs much more frequently associate with disease by either having subtle effects of disease predisposition or directly causing disease compared to the very few reports of beneficial control associated CNVs [31, 101, 102]. Therefore, having all identified CNVRs associate with case status is not unusual. Multiple other genes found in the CNVRs associated with ACL rupture influence cell cycle regulation, cellular signaling, apoptosis pathways, and translation/transcription regulation. Another interesting feature of the 46 associated CNVRs is that 12 of them do not contain genes that are annotated on the CanFam3.1 reference assembly. This may be due to poor annotation of the current canine genome assembly. Alternatively, these regions could be acting in a regulatory fashion to alter gene expression.

We determined that the standard cluster file provided by Illumina did not fit our Labrador Retriever population very well. The training sample set used to generate the commercially available cluster file represented over 20 diverse dog breeds. Creating a user-generated custom cluster file is recommended when samples are from a unique or isolated population [48, 49]. When CNV calling is performed from a SNP array, it is critical the cluster file accurately represents the data set, because the cluster positions are used to compute both LRR and BAF [49]. LRR and BAF are the essential input values which PennCNV [50], CNVPartition [51], and QuantiSNP [52] use to detect CNVs. Control samples were the only samples used to create the cluster file because cases may have a large amount of CNVs for biological reasons and those should not be included when defining cluster centroid positions [48]. Creating a breed-specific cluster file from the control samples with high call rate increased detection and accuracy of CNV calling.

Three algorithms were selected to process the same raw data set to produce a more comprehensive CNV analysis because of the known differences in CNV calling results produced by each analytic tool [103]. To our knowledge, there are no previous publications comparing and reporting performance of these three CNV algorithms in the dog. Given the underlying polygenic architecture of ACL rupture in the Labrador Retriever, we aimed to detect as many small subtle effect genetic contributions to disease risk without missing associations due to use of too few CNV calling algorithms. PennCNV, CNVPartition, and QuantiSNP were selected because together they offered an extensive CNV detection procedure although use of multiple algorithms may increase the likelihood of detecting false positives. Each program generated quality control variables that allowed for the exclusion of noisy samples. Published concordance rates of CNV calls from the same sample generated from any combination of two algorithms is 25–50% [104]. In this study, PennCNV and QuantiSNP (53.4%±15.3) had comparable concordance to QuantiSNP and CNVPartition (51.4%±15). However, PennCNV and CNVPartition had very low concordance (7.3%±9.4). Calling programs vary in the underlying mathematical models. An algorithm’s performance is influenced by the noise specific to that experiment, with factors such as the lab processing the sample, the array the samples are processed on, and the quality of the individual sample impacting results. Algorithm performance was evaluated by comparing the average CNVR length detected and comparing the overall base pair percentage coverage of the entire canine genome. With our data, CNVPartition called the least amount of CNVs, but those that were called were on average larger than the other algorithms. QuantiSNP called the most CNVs that were on average the shortest. Studies in humans and horses have reported PennCNV and QuantiSNP to have good concordance and similar amounts of CNVs calls, while CNVPartition has been reported to detect a small number of CNVs and have low concordance with PennCNV and QuantiSNP [105, 106]. This study suggests these algorithms perform similar in the dog as previously reported in other species.

Initially PennCNV was run without a GC correction. Many of the samples did not meet a waviness factor cut off of <-0.1 and >0.1. PennCNV and other programs have the ability to fit regression models with GC content to correct for variation in hybrid intensity that is related to the probe’s location in the genome. Genomic waves are not platform-specific and can prevent accurate CNV detection [107]. The waviness factor accounts for all of the signal fluctuation of a genotyped sample throughout the entire genome. The GC waviness factor measures the fraction of fluctuation that is explained by local GC content of the DNA. Including the GC content of the canine genome during calling greatly reduced the measured waviness factor for every sample and proved to be essential for accurate CNV calling of our data. GC correction was applied during CNV calling using PennCNV, CNVPartition, and QuantiSNP.

CNVRs containing genes found in the significant homeobox signaling pathway were evaluated with relative qPCR. Only a subset of the individuals with detected CNV deletions were able to be assessed because of limited sample DNA. Ambiguity exists in the interpretation of the qPCR results. The affected individuals did not produce expected relative copy numbers of 0 and 0.5 when homozygous and heterozygous deletions are present, respectively. Relative copy number estimated by qPCR in individuals with reported CNV called deletions ranged from 0.37–0.95. Canine CNV studies have reported two different categories of CNVRs. Canine CNVRs can be categorized as simple or complex based on the structural complexity of the CNVs comprising that region. Simple CNVRs have consistent patterns of breakpoints across individuals. Complex CNVRs have substantial variation in breakpoints between individuals along with spatial heterogeneity in copy number [38]. Within a particular complex CNVR, interesting patterns of alternating gains and losses have been observed. CNVs in dogs occurring in areas of segmental duplications are more likely to be complex [36, 108]. Segmental duplications are regions at least 90% identical at two or more loci that serve as hotspots of genomic rearrangement and CNV formation [38, 108]. One shortcoming of CNV calling using the CanineHD array is a decreased ability to identify short duplications/deletions due to the 13kb probe spacing [37]. Therefore, if there is a large complex CNVR that is comprised of smaller regions of alternating copy number state occurring consecutively, CNV calling and genotyping on an array with 13kb probe spacing may not correctly represent the complexity of that region [36]. Future investigation of these regions should include short-read whole genome sequencing (WGS) that provides higher resolution of CNV detection and breakpoint assessment. Because the chromosome 14 (*HoxA*) CNVR and chromosome 28 (*NKX6-2)* CNVR did not have shared CNV breakpoints amongst individuals, it provides evidence they are architecturally complex CNVRs. Having substantial variation between individual breakpoints or spatial heterogeneity in copy number would make qPCR validation challenging without precisely defining the fine-scale architectural complexity occurring at that location. A previous qPCR analysis of two CNVRs that were located in segmental duplications resulted in relative copy number changes that were not the expected 2 for a gain or 0.5 for a loss. Rather, smaller incremental relative changes were observed between individuals [38]. For example, if an individual with a loss when compared to a reference sample containing 3 copies would yield a relative copy number of 0.66. If the average copy number state for our Labrador Retriever population at these locations is greater than two copies, the qPCR results could be validating true relative signal reductions in the affected individuals. A previous canine CNV discovery paper reported a CNVR in the chromosome 28 (*NKX6-2)* CNVR locus providing support of a CNV present at this location [36]. None of the CNVRs found in the present study overlapped with previously reported canine ACL rupture risk loci. All detected CNVs were only associated at max(T) permutation test *P*<0.05, which suggests that CNV contribution to ACL rupture risk in the Labrador Retriever would not meet genome-wide significant thresholds during GWAS.

Major limitations with the supporting evidence regarding the ACL rupture associated homeobox developmental pathway are that the contributing genes are within CNVRs that were only detected by single calling algorithms and that the chromosome 36 (*HoxD*) and chromosome 14 (*HoxA*) regions have not been previously reported. Thirty four of the 46 ACL rupture associated CNVRs were only identified by a single calling algorithm. Despite stringent quality control methods, CNV detection from SNP array data has inherent limitations and not all CNVs called by one algorithm are confirmed with PCR analysis. Some CNVRs may be false positives despite use of rigorous methodology. Two affected individuals with reported *HoxD* CNVR deletions identified by a single calling algorithm were not validated with qPCR suggesting some dogs had deletion CNVs falsely called at this location. By running multiple algorithms on a single data set and using a nominal significance threshold (max(T) permutation *P*<0.05), together with FDR correction for CNVs called by multiple algorithms, the reported CNVRs are as comprehensive and inclusive as possible to help detect small subtle genetic contribution to a polygenic disease which may have subsequently increased the chance of reporting false positives.

Other limitations of this study include lack of correction for sex and neutering, body condition, and population stratification. ParseCNV does not account for environmental factors in the CNV association analysis. Without consideration of relevant covariates in the analysis, associated CNVRs may reflect candidate loci that predispose Labrador Retrievers to other confounding factors such as obesity, or knee morphology. Future work should include information collected on sex, age neutered, and body condition and appropriately incorporate them into analysis to avoid effects that might confound the association analysis. Interpretation of our results must be considered in light of the fact that ACL rupture is a complex polygenic disease and that only a single breed of dog was studied. qPCR validation of other CNVRs, particularly the 2 CNVRs on chromosomes 6 and 24 that had overlapping areas and were confirmed by all 3 algorithms could have been performed.

This work sets the stage for further validation and replication of ACL rupture associated CNVs. Because ambiguity exists between detection of duplications/deletions associated with cases in overlapping regions using the different algorithms, there is a need to confirm the presence and direction of a deviation in copy number from the reference genome. Further investigation of the fine-scale architecture of the homeobox pathway CNVRs is warranted. Short-read WGS has increased in popularity as a tool for CNV validation [109, 110] and could be used as a validation step in the Labrador Retrievers used in this study. Over time short-read WGS costs have decreased making it a more readily available high-throughput technology that allows researchers to perform detailed analysis of whole genomes. Short-read WGS is advantageous for CNV validation because it enables the detection of CNV presence, CNV structure, and determination of CNV breakpoints [111]. In addition, short-read WGS enables more detailed analysis of genomic loci identified by our GWAS and CNV analysis that will aid in revealing the genetic mechanisms influencing risk of ACL rupture. Analysis of a larger number of dogs for both femoral and tibial bony structural morphology would be helpful to examine the relationship between structural variation in genes that regulate skeletal patterning, limb morphology and associated risk of ACL rupture in more detail.

In conclusion, we identified 46 small effect CNVRs associated with ACL rupture in the Labrador Retriever in 39 unique regions of the genome. A total of 152 genes were located in associated CNVRs. Of these genes, 19 clustered into a significant homeobox biologic pathway. We provide evidence that homeobox genes could influence risk of ACL rupture in some dogs by influencing rTTW, an important feature of proximal tibial morphology. Going forward, the regions identified in this study should be validated using WGS to advance understanding of how CNVRs contribute to the genetic risk of ACL rupture. The relationship between associated CNVs and limb morphology also needs to be studied in more detail. Results of this work may aid in the creation of a genetic screening test for dogs at high genetic risk for ACL rupture and reveal further insight into the underlying biologic mechanisms that contribute to the etiology of the disease in both human beings and dogs.

## Supporting information

S1 File Programming commands used for PennCNV [50], QuantiSNP [52], and ParseCNV [53] for CNV calling and subsequent association analysis. (PDF)

S2 File Primers used for relative copy number qPCR. (PDF)

S3 File Examples of poor SNP clustering with the (a) standard cluster file compared to improved clustering with the (b) user-generated custom cluster file.
(PDF)
