# Supplementary material for: Analysis of copy number variation in dogs implicates genomic structural variation in the development of anterior cruciate ligament rupture
Source: PLoS One. 2020 Dec 31;15(12):e0244075. doi: 10.1371/journal.pone.0244075 (PMC7774950; doi:10.1371/journal.pone.0244075)
Supplement: S1 File — (PDF) [file pone.0244075.s001.pdf]

S1 File. Programming commands used for PennCNV [50], QuantiSNP [52], and ParseCNV [53] for CNV calling and subsequent association analysis.

PennCNV

PennCNV was run using the following commands:

```
>perl detect_cnv.pl –test –hmm lib/hhall.hmm –pfb pfbfile.pfb –listfile list.txt –lastchr 38 –log
sampleall.log –gcmodelfile gcmodelfile.gcmodel –conf – minsnp 3 –out sampleall.rawcnv
```

QuantiSNP

QuantiSNP was run using the following commands:

```
>quantisnp2 –verbose –outdir output –levels levels-hd.dat –config params.dat –logfile log
-beadstudio-files Beadstudiofile –gcdir canfam2gc –chr [1:38] –chrX 39 –genderfile gender.txt
```

ParseCNV

The quality controlled output for each of the three calling algorithms was separately analyzed using the following command in ParseCNV:

```
>perl ParseCNV.pl cases.rawcnv controls.rawcnv famfile.fam mapfile.map –build canFam2
–idToPath path.txt –permuteP 10000 –out output
```
