# Supplementary material for: Analysis of copy number variation in dogs implicates genomic structural variation in the development of anterior cruciate ligament rupture
Source: PLoS One. 2020 Dec 31;15(12):e0244075. doi: 10.1371/journal.pone.0244075 (PMC7774950; doi:10.1371/journal.pone.0244075)
Supplement: S2 File — (PDF) [file pone.0244075.s002.pdf]

S2 File. Primers used for relative copy number qPCR.

Primers used for each of the three homeobox CNVRs and an internal control (*C7orf28B*) [57]:

	Primer		Start bp	End bp
Chromosome 14<br>*HoxA* CNVR	Fwd.	TCCATCCTTTCTCTTCCTTTCC	40300286	40300307
	Rev.	ATTCGTTAGCTTCATCCCAGAG	40300366	40300387
Chromosome 28<br>*NKX6-2* CNVR	Fwd.	GACTAGCCTCAGAACCACATG	40514763	40514783
	Rev.	ATTCAGGACAGAACAGTGACG	40514882	40514902
Chromosome 36<br>*HoxD* CNVR	Fwd.	GTCAAATTCCCCTGCTTTTCC	19898551	19898571
	Rev.	GACACGCAAAACCCAGTAAC	19898620	19898639
Chromosome 6<br>*C7orf28B*	Fwd.	CAACACAGGTTGACCAAGGA	14333282	14333301
	Rev.	TTGTGCAGGATCAGAGCATC	14333211	14333230
