# Supplementary material for: Analysis of copy number variation in dogs implicates genomic structural variation in the development of anterior cruciate ligament rupture
Source: PLoS One. 2020 Dec 31;15(12):e0244075. doi: 10.1371/journal.pone.0244075 (PMC7774950; doi:10.1371/journal.pone.0244075)

S3 File. Examples of poor SNP clustering with the (a) standard cluster file compared to improved clustering with the (b) user-generated custom cluster file (b).

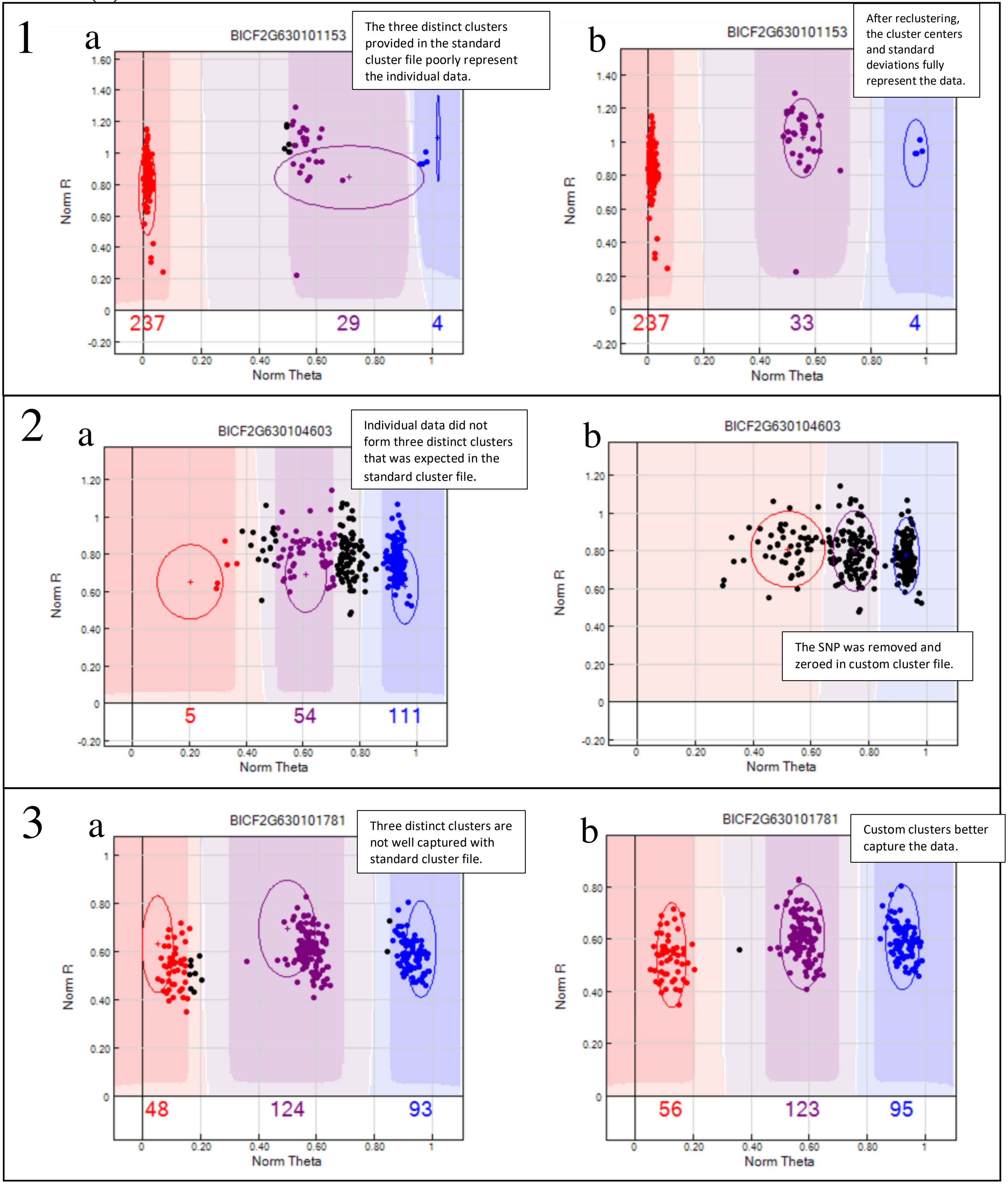

Supplement: S3 File — Examples of poor SNP clustering with the (a) standard cluster file compared to improved clustering with the (b) user-generated custom cluster file. (PDF) [file pone.0244075.s003.pdf]
